# Analyzing the composition of the editorial boards in high-impact medical ethics journals: a survey study

**DOI:** 10.1186/s12910-024-01006-2

**Published:** 2024-02-04

**Authors:** Zhiwei Jia, Donghua Liu, Xingxuan Li, Tianlin Wen, Xiyan Zhao, Wei Li

**Affiliations:** 1 Department of Orthopedics, Dongzhimen Hospital, Beijing University of Chinese Medicine, Beijing, China; 2 Department of Endocrinology, Guang’anmen Hospital, China Academy of Chinese Medical Sciences, Beijing, China; 3 Department of Sports Medicine, Fourth Medical Center of PLA General Hospital, Beijing, China

**Keywords:** Editorial board members, Medical ethics, Editor

## Abstract

**Background:**

The underrepresentation of scholarly works from low- and middle-income countries (LMICs) in academic literature is a documented concern, attributed partly to editorial biases. This trend, prevalent across various disciplines, has been less explored in the context of medical ethics journals. This study aimed to examine the composition of editorial board members (EBM) in high-impact medical ethics journals and to evaluate the extent of international diversity within these editorial teams.

**Methods:**

This study incorporated an analysis of 16 high-impact medical ethics journals. Information regarding the EBM of these journals was systematically gathered and categorized based on the World Bank’s country income classifications. An in-depth examination of the editorial board compositions was then conducted.

**Results:**

The study identified 669 EBM across the selected journals. A predominant 89.84% (601) of these members were from high-income countries (HICs), with upper-middle-income countries contributing 7.47% (50) and lower-middle-income countries 2.69% (18). No EBM were associated with low-income countries. A regional breakdown indicated that North America was the most represented area, accounting for 48.88% (327), followed by Europe & Central Asia (27.50%, 184), East Asia & Pacific (13.45%, 90), Latin America & Caribbean (4.63%, 31), Sub-Saharan Africa (4.19%, 28), Middle East & North Africa (0.75%, 5), and South Asia (0.60%, 4). In total, these EBMs hailed from 46 different countries, with the United States representing the largest proportion (43.80%, 293), followed by the United Kingdom (13.15%, 88), Australia (7.92%, 53), Germany (6.73%, 45), and Canada (5.08%, 34).

**Conclusions:**

There is a significant lack of international representation within the EBM of high-impact medical ethics journals. The majority of editors in this field are affiliated with HICs, leading to a severe underrepresentation of LMICs within the editorial boards.

## Introduction

Medical ethics, an integral component of the healthcare sector, plays a critical role in guiding professionals towards ethically sound decision-making and in safeguarding patient welfare and rights [1–3]. Tracing its origins to ancient civilizations and evolving continuously over centuries, medical ethics is foundational in ensuring the provision of ethical and compassionate patient care [4, 5]. This ongoing evolution is crucial to address the ethical challenges emerging from advancements in genetics and other biomedical/health technologies, as well as global healthcare issues [1, 4, 6, 7]. In particular, the ethical implications of emerging technologies, such as artificial intelligence and genomic medicine, necessitate continuous ethical scrutiny to guarantee their responsible and ethical application in patient care [6, 7].

In recent years, the development of medical ethics has exerted a substantial influence on the healthcare landscape [1, 3, 7]. The dissemination of medical ethics research is pivotal in informing ethical decision-making in both patient care and biomedical research [2–5]. Countries contribute variably to medical advancements, influenced by their unique social, economic, and medical contexts [8–10]. Researchers from low- and middle-income countries (LMICs), which constitute a significant portion of the global population and bear a considerable disease burden, frequently face challenges in publishing their work in esteemed medical journals [11–13]. Factors such as restricted funding, inexperienced researchers, and language barriers are known to hinder research productivity in LMICs [12, 14–19]. Additionally, editorial bias has been identified as a key factor in the underrepresentation of publications from LMICs [13–15, 20–25].

Researchers in LMICs often perceive significant barriers to publishing in high-impact journals [12, 13]. Studies indicate that editorial bias may contribute to the low proportion of papers from authors affiliated with LMICs [14, 26, 27]. The composition of editorial board members (EBM), which shapes the personality, policy, and preferred content of journals, can introduce inherent biases [14, 28, 29]. The cultural predominance of high-income countries (HICs) potentially influences the focus and content of these journals, often leading to a preponderance of publications concerning conditions prevalent in HICs and fewer studies addressing healthcare issues in LMICs [30–33]. Evidence suggests that a more diverse representation in editorial boards correlates with increased publications from LMICs in leading biomedical journals [34].

Assessing the international representation within editorial teams, specifically the composition of EBM in prominent medical ethics journals, is thus imperative [14–16, 35]. Previous studies have highlighted a significant underrepresentation of EBM from LMICs across various medical fields [14, 15, 26, 28–31], yet the extent of this phenomenon within medical ethics journals remains underexplored. Therefore, the present study aimed to analyze the EBM composition in major medical ethics journals and shed light on the international representation of editorial staff in this crucial domain of medical ethics.

## Methods

This research employed a content analysis of journal websites and was exempt from Institutional Review Board approval due to the absence of human or animal subjects. The research methodology employed in this study was based on similar publications within the disciplines of paediatrics, psychiatry, foot and ankle surgery, and spine [14, 15, 28, 29]. For the identification of relevant medical ethics journals, the Journal Citation Reports from 2021 were consulted, culminating in the selection of sixteen high-impact journals, as detailed in Table 1.

**Table 1:** List of high-impact medical ethics journals

Journal title	Abbreviation	Country of publication	Impact factor
*American Journal of Bioethics*	AJB	United States	14.676
*Journal of Law and the Biosciences*	JLB	United States	6.066
*Journal of Medical Ethics*	JME	England	5.926
*Hastings Center Report*	HCR	United States	4.298
*Accountability in Research-Policies and Quality Assurance*	ARPQA	United States	3.057
*BMC Medical Ethics*	BME	England	2.834
*Public Health Ethics*	PHE	England	2.706
*Bioethics*	BE	England	2.512
*Developing World Bioethics*	DWB	England	2.427
*Journal of Bioethical Inquiry*	JBI	New Zealand	2.216
*Philosophy Ethics and Humanities in Medicine*	PEHM	England	2.200
*Journal of Empirical Research on Human Research Ethics*	JERHRE	United States	1.978
*Journal of Law Medicine & Ethics*	JLME	United States	1.604
*Neuroethics*	NE	Netherlands	1.427
*Ethik in der Medizin*	EM	Germany	0.729
*Acta Bioethica*	AB	Chile	0.490

Data collection took place on May 5, 2023, involving a review of the official websites of the chosen journals. The study focused on gathering and analyzing data related to the number of EBM and their countries of origin. The geographical distribution of EBM was systematically categorized into seven regions, as per the World Bank classification (www.worldbank.org): Europe & Central Asia (ECA), North America (NA), East Asia & Pacific (EAP), Latin America & Caribbean (LAC), Middle East & North Africa (MENA), South Asia (SA), and Sub-Saharan Africa (SSA). Additionally, the World Bank’s income group classification system was utilized to further categorize countries into low-, lower-middle-, upper-middle-, and high-income groups based on their Gross National Income per capita.

Criteria for identifying major countries represented by EBM included a threshold wherein the number of members constituted at least 1% of the global editorial representation in medical ethics journals [36, 37]. The representation from each country was then standardized relative to its population size and gross domestic product (GDP). Furthermore, the study explored the correlation between the number of published papers and the population and GDP of each country, sourcing this data from the World Bank.

It is essential to emphasize that the primary aim of this study was to elucidate trends and provide descriptive statistics, rather than to test hypotheses about the impact of geographic diversity in EBM on submissions and publications in medical ethics journals. To this end, descriptive statistical methods, including the calculation of sums and proportions, were predominantly applied in the analysis. The global distribution of EBM was visualized using MapChart (www.mapchart.net), which allows any map generated to be freely used, edited, and modified for private, commercial, and public purposes.

## Results

**Fig. 1:**
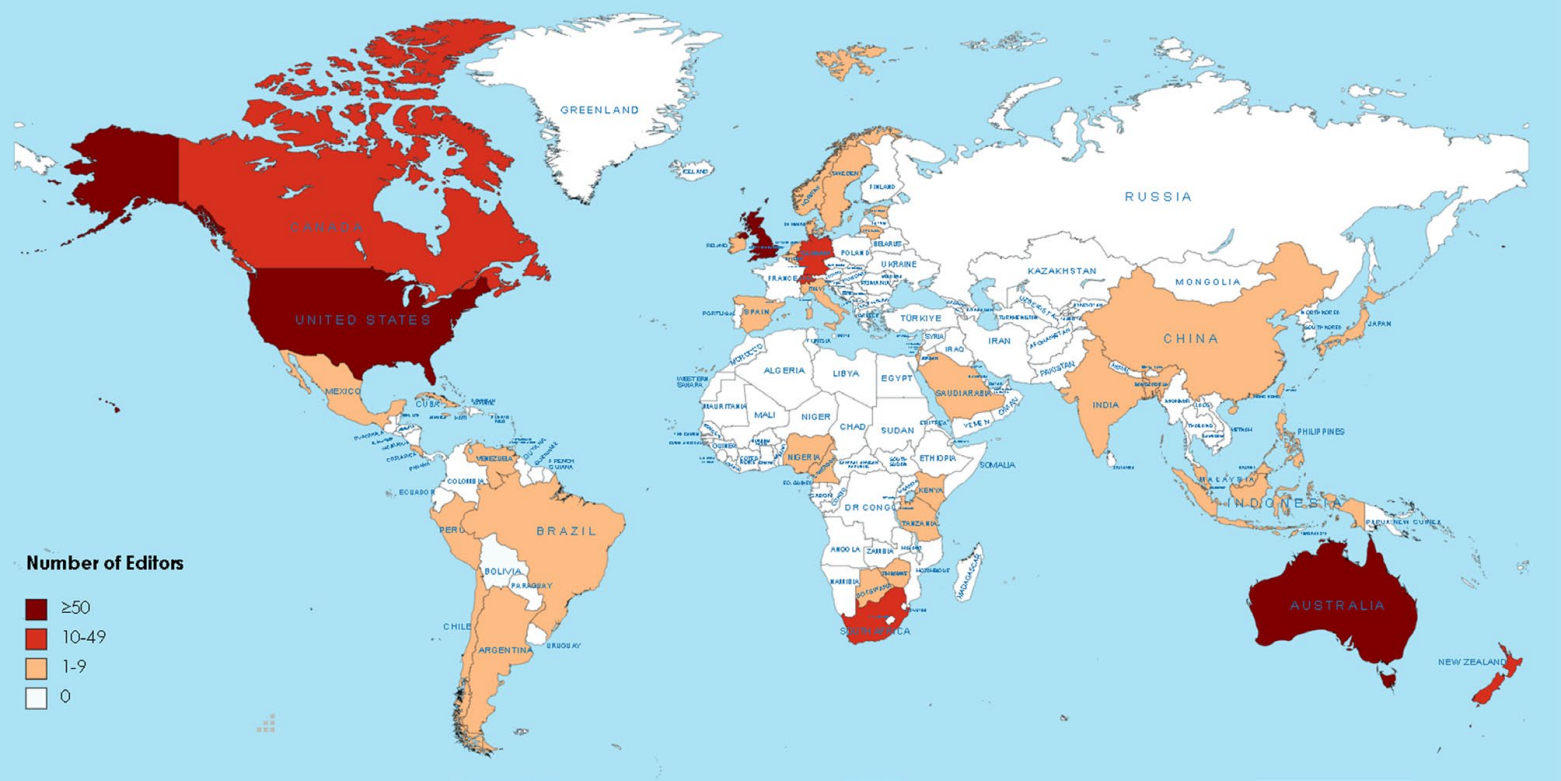
The world distributions of editorial board members. The world map was created with MapChart (www.mapchart.net)

In this analysis of the 16 medical ethics journals, a total of 669 EBM were identified. These members were from 46 countries, comprising 24 HICs, 14 upper-middle-income countries (UMICs), and 8 lower-middle-income countries. Figure 1 illustrates the geographic distribution of EBM, with the United States having the highest number (293, 43.80%), followed by the United Kingdom (88, 13.15%), Australia (53, 7.92%), Germany (45, 6.73%), and Canada (34, 5.08%).

Regarding the origins of the selected journals, six (37.5%) were based in the United States, including *American Journal of Bioethics* (AJB), *Journal of Law and the Biosciences* (JLB), *Hastings Center Report* (HCR), *Accountability in Research-Policies and Quality Assurance* (ARPQA), *Journal of Empirical Research on Human Research Ethics* (JERHRE), and *Journal of Law Medicine & Ethics* (JLME). Another six (37.5%) were from England, comprising *Journal of Medical Ethics* (JME), *BMC Medical Ethics* (BME), *Public Health Ethics* (PHE), *Bioethics* (BE), *Developing World Bioethics* (DWB), and *Philosophy Ethics and Humanities in Medicine* (PEHM). The remaining journals included one each from New Zealand (6.25%) (*Journal of Bioethical Inquiry*, JBI), the Netherlands (6.25%) (*Neuroethics*, NE), Germany (6.25%) (*Ethik in der Medizin*, EM), and Chile (6.25%) (*Acta Bioethica*, AB).

The regional distribution of EBM across these journals, presented in Table 2, shows that NA accounts for the largest share with 48.9%, followed by ECA at 27.5%, EAP at 13.5%, LAC at 4.6%, SSA at 4.2%, MENA at 0.7%, and SA at 0.6%. Notably, NA was the predominant region in most journals, with AJB having the highest proportion of NA-based editors (98.1%), followed by JLME (95.8%) and HCR (94.1%). EM had all its editors (100%) from ECA, with JME (57.8%) and BE (52.8%) also having significant representation from this region. JBI had the highest proportion of editors (45.7%) from EAP, followed by JME (24.4%) and PHE (18.4%).

**Table 2:** The editorial board members classified by regions

Journal title	North America	Europe & Central Asia	East Asia & Pacific	Latin America & the Caribbean	Middle East and North Africa	South Asia	Sub-Saharan Africa
*American Journal of Bioethics*	98.1%	1.9%	0.0%	0.0%	0.0%	0.0%	0.0%
*Journal of Law and the Biosciences*	82.9%	11.4%	5.7%	0.0%	0.0%	0.0%	0.0%
*Journal of Medical Ethics*	17.8%	57.8%	24.4%	0.0%	0.0%	0.0%	0.0%
*Hastings Center Report*	94.1%	5.9%	0.0%	0.0%	0.0%	0.0%	0.0%
*Accountability in Research-Policies and Quality Assurance*	72.7%	9.1%	15.2%	0.0%	0.0%	0.0%	3.0%
*BMC Medical Ethics*	25.5%	45.5%	16.4%	1.8%	1.8%	0.0%	9.1%
*Public Health Ethics*	40.8%	30.6%	18.4%	2.0%	0.0%	2.0%	6.1%
*Bioethics*	32.1%	52.8%	11.3%	0.0%	1.9%	0.0%	1.9%
*Developing World Bioethics*	18.9%	8.1%	8.1%	27.0%	2.7%	8.1%	27.0%
*Journal of Bioethical Inquiry*	30.9%	16.0%	45.7%	1.2%	1.2%	0.0%	4.9%
*Philosophy Ethics and Humanities in Medicine*	88.9%	0.0%	0.0%	0.0%	0.0%	0.0%	11.1%
*Journal of Empirical Research on Human Research Ethics*	79.2%	8.3%	4.2%	0.0%	2.1%	0.0%	6.3%
*Journal of Law Medicine & Ethics*	95.8%	0.0%	4.2%	0.0%	0.0%	0.0%	0.0%
*Neuroethics*	52.1%	35.4%	10.4%	2.1%	0.0%	0.0%	0.0%
*Ethik in der Medizin*	0.0%	100.0%	0.0%	0.0%	0.0%	0.0%	0.0%
*Acta Bioethica*	18.2%	4.5%	0.0%	77.3%	0.0%	0.0%	0.0%
Total	48.9%	27.5%	13.5%	4.6%	0.7%	0.6%	4.2%

When categorized by income groups, as detailed in Table 3, it was found that all EBM in AJB, JLB, HCR, JLME, and EM were from HICs. Overall, HICs were represented by 89.8% of the EBM, UMICs by 7.5%, and lower-middle-income countries by 2.7%. No EBM from low-income countries were identified.

**Table 3:** The editorial board members classified by income group

Journal title	High-income countries	Upper-middle-income countries	Lower-middle-income countries	Low-income countries
*American Journal of Bioethics*	100.0%	0.0%	0.0%	0.0%
*Journal of Law and the Biosciences*	100.0%	0.0%	0.0%	0.0%
*Journal of Medical Ethics*	97.8%	2.2%	0.0%	0.0%
*Hastings Center Report*	100.0%	0.0%	0.0%	0.0%
*Accountability in Research-Policies and Quality Assurance*	93.9%	6.1%	0.0%	0.0%
*BMC Medical Ethics*	89.1%	1.8%	9.1%	0.0%
*Public Health Ethics*	87.8%	8.2%	4.1%	0.0%
*Bioethics*	96.2%	3.8%	0.0%	0.0%
*Developing World Bioethics*	32.4%	45.9%	21.6%	0.0%
*Journal of Bioethical Inquiry*	88.9%	7.4%	3.7%	0.0%
*Philosophy Ethics and Humanities in Medicine*	88.9%	11.1%	0.0%	0.0%
*Journal of Empirical Research on Human Research Ethics*	93.8%	6.3%	0.0%	0.0%
*Journal of Law Medicine & Ethics*	100.0%	0.0%	0.0%	0.0%
*Neuroethics*	97.9%	2.1%	0.0%	0.0%
*Ethik in der Medizin*	100.0%	0.0%	0.0%	0.0%
*Acta Bioethica*	45.5%	54.5%	0.0%	0.0%
Total	89.8%	7.5%	2.7%	0.0%

An analysis of major contributing countries, outlined in Table 4, identified 13 countries across various regions, including ECA (4), EAP (4), NA (2), LAC (2), and SSA (1). The majority were HICs (10), with three being UMICs. Upon standardizing the number of EBM relative to the population sizes and GDPs of their respective countries, New Zealand, Australia, and Switzerland emerged as top contributors. When GDP was considered, New Zealand, South Africa, and Australia were notably prominent.

**Table 4:** The major countries of editorial board members in high-impact medical ethics journals

Rank	Countries	Region	Income Group	No. of Editorial Staff	Percentage	No. per 10 Million People (Rank)	No. per $ 1000 Billion GDP (Rank)
1	United States	NA	HICs	293	43.80%	8.8 (6)	12.7 (9)
2	United Kingdom	ECA	HICs	88	13.15%	13.1 (4)	27.6 (4)
3	Australia	EAP	HICs	53	7.92%	20.6 (2)	34.4 (3)
4	Germany	ECA	HICs	45	6.73%	5.4 (7)	10.7 (10)
5	Canada	NA	HICs	34	5.08%	8.9 (5)	17.1 (7)
6	South Africa	SSA	UMICs	17	2.54%	2.8 (10)	40.5 (2)
7	Switzerland	ECA	HICs	15	2.24%	17.2 (3)	18.5 (6)
8	New Zealand	EAP	HICs	12	1.79%	23.4 (1)	48.0 (1)
9	Netherlands	ECA	HICs	9	1.35%	5.1 (8)	8.8 (11)
10	Japan	EAP	HICs	7	1.05%	0.6 (12)	1.4 (12)
11	China	EAP	UMICs	7	1.05%	0.0 (13)	0.4 (13)
12	Chile	LAC	HICs	7	1.05%	3.6 (9)	22.1 (5)
13	Argentina	LAC	UMICs	7	1.05%	1.5 (11)	14.2 (8)

NA, North America; EAP, East Asia & Pacific; ECA, Europe & Central Asia; LAC, Latin America & Caribbean; SSA, Sub-Saharan Africa; HICs, High-income countries; UMICs, Upper-middle-income countries

## Discussion

The advancement of medical ethics research globally is profoundly influenced by contributions from researchers around the world [1, 2, 4, 5]. The publication of new findings is a pivotal aspect of research activities [3, 6, 7, 38]. Editorial boards, as central entities of academic journals, exert considerable influence over the publication landscape and the future direction of these journals [26, 30, 31]. However, it has been observed that a disproportionately low number of publications originate from LMICs in multiple medical journals [10, 11, 32, 33, 39, 40]. This discrepancy can be attributed to factors such as limited financial resources, inadequate research infrastructures, and language barriers [18, 28, 29]. Additionally, editorial bias, manifesting as unfavorable treatment towards submissions from LMICs by journal editorial boards, has been a subject of concern [14, 15, 26, 28–31]. Enhancing the diversity of editorial staff members is crucial to broaden peer review perspectives and encourage submissions from researchers of diverse backgrounds [16, 17, 20, 23–25, 35]. Yet, an underrepresentation of editorial staff from LMICs persists across disciplines including pediatrics, psychiatry, foot and ankle surgery, and spine, as well as in medical education and anesthesiology/critical care [14, 15, 26, 28–31]. However, there is a lack of investigation into the composition of EBM specifically in high-impact medical ethics journals [41].

Our findings indicate that the composition of EBM in medical ethics journals is predominantly concentrated in a few countries, notably the United States, the United Kingdom, Australia, and Germany. Collectively, these countries account for over 70% of total EBM, thereby significantly shaping the identity and editorial policies of these journals, especially into those that reflect publications from the United States [14, 15, 28, 29]. This concentration suggests that editors from underrepresented nations may have limited impact on the published content of these journals [26, 31, 34, 42]. The affiliation of EBM is instrumental in setting the journals’ priorities and influencing their scientific output [29, 34, 43, 44]. Research has shown that greater diversity in editorial boards correlates with increased publication of work from LMICs in leading biomedical journals [34]. Editors from underrepresented regions may wield diminished influence in several critical aspects of scholarly publishing. This includes the scope of topics deliberated, the development and application of conceptual frameworks, decision-making regarding journal priorities, and the process of article acceptance. Their limited participation can lead to a narrowed perspective within the journal, potentially skewing the academic discourse away from a more globally inclusive and diverse viewpoint. This imbalance underscores the necessity of ensuring equitable representation on editorial boards to enrich and diversify the intellectual dialogue and decision-making processes within academic journals [14, 15, 27, 28, 34].

The distribution of editors is uneven globally, with NA, ECA, and EAP comprising nemajorarly 90% of total editors. This imbalance may be attributed to the fact that leading countries with the highest research output are affiliated with these regions, resulting in a higher number of editors from these countries. Recognizing this imbalance, editorial boards must address the under-representation of editors from other regions [26, 31, 42].

In high-impact medical ethics journals, approximately 90% of editors are affiliated with HICs, with a marginal representation from middle-income countries and none from low-income countries. This under-representation of editors from LMICs is consistent with observations in several fields, including paediatrics, psychiatry, foot and ankle surgery, spine, anesthesiology/critical care, and hand surgery [14, 15, 26, 28, 29, 31, 45]. The underrepresentation of LMIC editors can significantly affect bioethics discourse, potentially leading to less attention on medical ethics issues prevalent in LMICs and matters of scarce resource allocation [46, 47]. Moreover, when normalized by their larger populations, the relative percentage of editors from LMICs appears even lower, as evident in our study [14, 15].

Among high-impact medical ethics journals, six journals (AJB, JLB, HCR, ARPQA, JERHRE, and JLME) are affiliated with the United States, eight with Europe, one with EAP, and one with LAC. It is noteworthy that the majority of editors in AJB, JLB, HCR, ARPQA, JERHRE, and JLME are affiliated with NA, those in JME, BME, BE, and EM are affiliated with Europe, those in JBI are affiliated with EAP, and those in AB are affiliated with LAC. This indicates a tendency for international medical ethics journals to appoint editors from their respective regions, an aspect that should be considered by these journals [14, 15].

While our study analyzes the composition of editors in high-impact medical ethics journals, the question of editorial bias in the field of medical ethics research remains, despite the low representation of editors from LMICs. The inclusion of diverse editors is likely to promote varied and balanced perspectives [14, 15, 23, 35]. However, the current imbalance may contribute to an inherent bias, possibly resulting in a greater focus on issues pertinent to HICs and less emphasis on healthcare challenges in LMICs [14, 15, 26, 27, 30, 31, 48, 49]. Furthermore, there are issues related to the underrepresentation of LMIC voices in bioethics discussions. First, perspectives from researchers in LMICs on emerging health/biomedical technologies, including artificial intelligence and genomic medicine, may be underrepresented [6, 7]. Second, there is a potential lack of discourse on ethical issues surrounding diseases that predominantly affect LMICs, such as spinal cord injuries [47]. Third, certain philosophical/moral frameworks are less likely considered, particularly in discussions on topics like end-of-life care [50, 51]. Most importantly, a critical aspect of the overrepresentation in bioethics research from HICs is the potential oversight of significant ethical issues that are prevalent in LMICs. An illustrative example is the realm of pediatric kidney failure care in LMICs, which presents a spectrum of ethical challenges distinct from those encountered in HICs. Research indicates that children with kidney failure in LMICs face considerable disparities, such as limited access to maintenance dialysis, timely kidney transplantation, and palliative care, compared to their counterparts in HICs [46]. These disparities underscore the necessity for the global pediatric nephrology community to recognize and address the unique ethical dilemmas arising in resource-constrained settings. Nephrologists in LMICs often grapple with complex decision-making scenarios for children with kidney failure, situations that are compounded by the constrained healthcare resources available. The disparity in treatment options and healthcare infrastructure between LMICs and HICs not only highlights significant ethical challenges but also underscores the pressing need for a more inclusive and representative bioethics discourse that duly considers the varied contexts and resources available globally [38, 46, 47].

Addressing the lack of LMIC representation in medical ethics journals is imperative. Journals and the medical ethics research community should take proactive measures to mitigate potential biases [11, 14, 15, 23, 32, 35]. Achieving a balanced composition of editors from diverse regions and income groups is essential for the advancement of medical ethics research [14, 15, 23, 31, 35]. This might involve appointing more editors from LMICs and implementing rotational policies for editors from different countries [14, 15, 26–31]. However, there are challenges for EBM from LMICs in academic journals. Since most leading medical ethics journals are published in English, EBM must be proficient in English, which can be a barrier for many in non-English speaking LMICs [28, 29, 34, 45]. Additionally, researchers from LMICs may lack the extensive experience required for high-level editorial roles in medical ethics journals [28, 29, 45].

Our study has limitations, including potential language bias due to the inclusion of journals published primarily in English, German, and Spanish [12, 28]. Moreover, the limited number of journals analyzed may affect the generalizability of our findings [14, 15, 28, 29]. Nonetheless, the 16 high-impact medical ethics journals examined are representative of major international publications in the field.

## Conclusion

There is a noticeable lack of international representation among EBM in high-impact medical ethics journals. The majority of editors are affiliated with HICs, leading to a severe underrepresentation of LMICs in the field of medical ethics.

## Data Availability

The data that support the findings of this study are available from the corresponding author upon reasonable request.

## Abbreviations

AB: Acta Bioethica
AJB: American Journal of Bioethics
ARPQA: Accountability in Research-Policies and Quality Assurance
BE: Bioethics
BME: BMC Medical Ethics
DWB: Developing World Bioethics
EAP: East Asia & Pacific
EBM: Editorial board members
ECA: Europe & Central Asia
EM: Ethik in der Medizin
GDP: Gross domestic product
HCR: Hastings Center Report
HICs: High-income countries
JBI: Journal of Bioethical Inquiry
JERHRE: Journal of Empirical Research on Human Research Ethics
JLB: Journal of Law and the Biosciences
JLME: Journal of Law Medicine & Ethics
JME: Journal of Medical Ethics
LAC: Latin America & Caribbean
LMICs: Low- and middle- income countries
MENA: Middle East & North Africa
NA: North America
NE: Neuroethics
PEHM: Philosophy Ethics and Humanities in Medicine
PHE: Public Health Ethics
SA: South Asia
SSA: Sub-Saharan Africa
UMICs: Upper-middle-income countries
